# LncRP11-675F6.3 responds to rapamycin treatment and reduces triglyceride accumulation via interacting with HK1 in hepatocytes by regulating autophagy and VLDL-related proteins: LncRNA with HK1 regulates triglycerides, autophagy and VLDL

**DOI:** 10.3724/abbs.2023091

**Published:** 2023-05-24

**Authors:** Lingling Wang, Xiaojuan Fang, Ziyou Yang, Xueling Li, Mengdi Cheng, Liang Cheng, Ganglin Wang, Wei Li, Lin Liu

**Affiliations:** 1 Key Laboratory of Laboratory Medicine Ministry of Education of China Zhejiang Provincial Key Laboratory of Medical Genetics School of Laboratory Medicine and Life Sciences Wenzhou Medical University Wenzhou 325035 China; 2 Zhuji Affiliated Hospital of Wenzhou Medical University Shaoxing 311800 China

**Keywords:** lncRP11-675F6.3, triglycerides, autophagy, lipoprotein, hexokinase 1, nonalcoholic fatty liver disease

## Abstract

Long noncoding RNAs (lncRNAs) have been widely proven to be involved in liver lipid homeostasis. Herein, we identify an upregulated lncRNA named *lncRP11-675F6.3* in response to rapamycin treatment using a microarray in HepG2 cells. Knockdown of *lncRP11-675F6*. *3* leads to a significant reduction in apolipoprotein 100 (ApoB100), microsomal triglyceride transfer protein (MTTP), ApoE and ApoC3 with increased cellular triglyceride level and autophagy. Furthermore, we find that ApoB100 is obviously colocalized with GFP-LC3 in autophagosomes when *lncRP11-675F6*. *3* is knocked down, indicating that elevated triglyceride accumulation likely related to autophagy induces the degradation of ApoB100 and impairs very low-density lipoprotein (VLDL) assembly. We then identify and validate that hexokinase 1 (HK1) acts as the binding protein of *lncRP11-675F6.3* and mediates triglyceride regulation and cell autophagy. More importantly, we find that *lncRP11-675F6.3* and HK1 attenuate high fat diet induced nonalcoholic fatty liver disease (NAFLD) by regulating VLDL-related proteins and autophagy. In conclusion, this study reveals that *lncRP11-675F6.3* is potentially involved in the downstream of mTOR signaling pathway and the regulatory network of hepatic triglyceride metabolism in cooperation with its interacting protein HK1, which may provide a new target for fatty liver disorder treatment.

## Introduction

Lipid metabolism in the liver plays essential roles in the homeostasis of nutrients and energy [1]. In general, the metabolic process of lipids in hepatocytes includes uptake of fatty acids, *de novo* lipogenesis, lipolysis, β-oxidation, lipid droplet formation and release of very low-density lipoproteins (VLDL) [2]. Aberrant lipid metabolism in the liver forms the basis for many metabolic disorders, such as hyperlipidemia, steatosis, insulin resistance and cholelithiasis [3]. Therefore, drug development for metabolic diseases naturally targets the molecules involved in the metabolic pathways in the liver. For instance, statins, as HMG-CoA reductase inhibitors, are the best-known drugs for the treatment of hypercholesterolemia [4].

With the increasing prevalence of nonalcoholic fatty liver disease (NAFLD), it has become imperative to discover drugs for NAFLD [5]. At present, the putative targets range from lipid homeostasis to antioxidant stress, mitochondrion regulation, and crosstalk with mediators related to hepatic macrophages and stellate cells [6]. Several candidates that are used in ongoing clinical trials include PPAR agonists, FXR agonists, THRβ agonists, galectin-3 inhibitors and SCD1 inhibitors [7]. However, none has been approved for clinical use to date. Therefore, further investigation of the pivotal nodes of the regulatory network in lipid metabolism is a prerequisite for the development of novel drug targets.

Long noncoding RNAs (lncRNAs) function as signals, decoys, guides, and scaffolds and have been extensively reported to be involved in gene regulatory networks [8], including lipid metabolism in hepatocytes [9]. Several lncRNAs have been well demonstrated to be associated with critical transcription factors regulating cholesterol and triglycerides, such as *LeXis*, which is a lipid- or LXR-responsive noncoding RNA that interacts with Raly and affects the DNA interactions of RALY to modulate cholesterol metabolism in the mouse liver [10]. *LncRNA H19* increases the transcriptional activity of SREBP-1c by promoting its mRNA binding to PTBP1. Ablation of *H19* alleviates high-fat diet-induced steatosis [11]. *Blnc1* is necessary for the induction of SREBP1c and hepatic lipogenic genes in response to LXR activation. Liver-specific silencing of *Blnc1* reduces high fat diet-induced steatosis and protects mice from nonalcoholic steatohepatitis (NASH) [12].

Some lncRNAs regulating lipid metabolism could affect the gene expressions of lipoproteins. *LncLSTR* interacts with TDP-43 and negatively regulates *Cyp8b1*, which inhibits *apoC2* gene expression through FXR. Therefore, *LncLSTR* depletion elevates apoC2 expression, resulting in lipoprotein lipase activation and increased plasma triglyceride clearance [13]. Long noncoding antisense RNA *APOA1-AS* functions as a negative transcriptional regulator of *APOA1*, and silencing of *APOA1-AS* results in increased expression of *APOA1* and two neighboring genes in the APO cluster [14]. Another antisense lncRNA, *APOA4-AS*, directly interacts with HuR to stabilize *APOA4* mRNA. Knockdown of *APOA4-AS* leads to a reduction in *APOA4* expression and decreased levels of plasma triglycerides and total cholesterol in mice [15]. It was reported in FANTOM5 that there are nearly 20 thousands long noncoding RNAs detected in the human genome [16]. The functions of the great majority of lncRNAs remain unclear.

In the present study, we identified a novel lncRNA named *lncRP11-675F6.3* that could interact with HK1 and attenuate lipid accumulation in hepatocytes *in vitro* and *in vivo* by regulating autophagy and VLDL-related proteins, which may provide a new therapeutic strategy by delivering lncRNAs to the liver.

## Materials and Methods

### siRNA oligos and vectors

siRNA oligos for both *lncRP11-675F6.3* and HK1 as well as negative controls were designed and synthesized by RiboBio (Guangzhou, China). All siRNA oligos' sequences are listed in Supplementary Table S1. The plasmid pBABEpuro GFP-LC3 was obtained from Addgene (#22405; Watertown, USA). The lentiviral transfer plasmid pCDH-EF1-MCS (System Biosciences, Palo Alto, USA) was used to carry full-length *lncRP11-675F6.3* cDNA. Lentiviral particles with a final titter of 10 ^12^ Pfu/mL were generated by Weizhen Bioscience (Ji’nan, China). Adeno-associated virus (AAV) vectors carrying either the lncRP11-675F6.3 or HK1 cDNA sequence as well as the GFP negative control were also generated by Weizhen Bioscience.

### Cell culture

Cell lines, including HepG2, Huh7 and HL-7702 (LO2), were all obtained from the Cell Bank of the Chinese Academy of Sciences (Shanghai, China). Both HepG2 and Huh7 cells were maintained in Dulbecco’s modified Eagle’s medium (DMEM; Invitrogen, Grand Island, USA) supplemented with 10% fetal bovine serum (FBS; Ginimi, Woodland, USA). HL-7702 cells were cultured in RPMI-1640 medium (GE Healthcare, Little Chalfont, UK) supplemented with 10% FBS. Cell cultures were all maintained in a humidified incubator at 37°C with 5% CO _2_. For mTOR inhibition, HepG2 cells were treated with rapamycin (Selleck Chemical, Houston, USA) at a final concentration of 20 nM for 8 h, and 0.1% DMSO was used as a negative control. For the lipid test, HepG2 cells were treated with oleic acid (OA; Sigma, St Louis, USA) at the indicated concentrations for 24 h after siRNA transfection. siRNA transfection in HepG2 cells was performed using Lipofectamin3000 (Life Technology, Carlsbad, USA) according to the manufacturer’s protocol and routine operation in our own laboratory. Autophagy inhibitors, either chloroquine (CQ; Sigma) at 4 μM or 3-methyladenine (3-MA; Med Chem Express, Shanghai, China) at 1 mM, were used to treat HpeG2 cells for 12 h after siRNA transfection. Lentiviral particle transfection of HepG2 cells was performed according to the previous protocol [17] to achieve stable *lncRP11-675F6.3* overexpression.

### Animals

C57BL/6J mice were obtained from the Zhejiang Vital River Laboratory Animal Technology Company (Jiaxing, China) and housed in the Experimental Animal Center of Wenzhou Medical University. All animal experiments were performed in accordance with the recommendations in the Guide for the Care and Use of Laboratory Animals of the National Institutes of Health and in line with the Global 3R Initiative. All protocols were approved by the Committee on the Ethics of Animal Experiments of Wenzhou Medical University (No. xmsq 2021-0079).

Mice were acclimatized to the housing conditions for 7 days and were then randomly assigned into 5 groups (8 mice per group) that matched for age, gender and weight. One group was fed a chow diet as a normal control, and the others were fed a 45% high fat diet (HFD; Medicience, Yangzhou, China). The HFD groups were injected via the tail vein once a week with 200 μL of saline, AAV-GFP, AAV-lncRP11-675F6.3 and AAV-HK1, respectively. All groups were maintained for 9 weeks, and body weight was measured each week. At the end of experiment, mice were euthanized with 2% sodium pentobarbital and subjected to euthanasia. The livers were harvested and frozen in liquid nitrogen for further analysis.

### LncRNA microarray and bioinformatics analysis

Total RNA was extracted from HepG2 cells using Trizol reagent (Invitrogen). LncRNA profiling was analyzed using LncRNA+mRNA Human Gene Expression Microarray V4.0, 4x180K (Capitalbio, Beijing, China). The microarray contains 77,000 lncRNA probes assembled from databases such as ENSEMBL, HumanLincRNACatalog, RefSeq and other related literature. After hybridization, the processed slides were scanned, and subsequently the raw data were extracted as pair files by Feature Extraction software. The gene expression differences and *P* values were then calculated by GeneSpring GX software. The threshold set for upregulated and downregulated lncRNAs was a fold change ≥1.5 and a *P* value≤0.05. Hierarchical clustering of the lncRNA profiles was performed using Cluster 3.0.

LncRNA gene chromosome location and sequence alignment were performed using the Human BLAT Search Tool ( https://genome.ucsc.edu/). The protein-coding potential of the lncRNA sequences was evaluated by Coding Potential Calculator (CPC; http://cpc.cbi.pku.edu.cn/programs/run_cpc.jsp) and Coding Potential Assessment Tool (CPAT, http://lilab.research.bcm.edu/index.php). LncRNA secondary structure prediction was obtained through the RNAfold web server ( http://rna.tbi.univie.ac.at/cgi-bin/RNAWebSuite/RNAfold.cgi).

### RNA ligase-mediated rapid amplification of cDNA ends (RLM-RACE)

Primers for 5′ and 3′RACE were designed according to the sequence from the UCSC genome and synthesized by Sangon Biotech (Shanghai, China). The sequences are listed in Supplementary Table S2. RLM-RACE was performed using the kit (#AM1700; Ambion, Austin, USA) according to the manufacturer’s protocol. Briefly, CIP treatment was first used for 5′ RLM-RACE to remove 5′ PO4 from degraded RNA, then TAP treatment was used to remove cap from full-length RNA and 5′RACE adaptor ligated to decapped RNA, subsequently reverse transcription and PCR were performed using lncRNA specific primer and 5′ adaptor primer. For 3′RACE, reverse transcription was first conducted using poly-T-containing adaptor primers, and then PCR was performed using specific primers and 3′ adaptor primers. Both 5′ and 3′ RLM-RACE PCR products were cloned into the pMD19-T vector (TaKaRa, Tokyo, Japan) and submitted for sequencing. The reagents and kits used in RLM-RACE were the SuperScriptIII First-Strand Synthesis System (Invitrogen), *Taq* DNA polymerase (TaKaRa), Gel Extraction kit (Omage, Norcross, USA), and pMD19-T vector (TaKaRa), respectively.

### Fluorescence test

Fluorescence *in situ* hybridization (FISH) was carried out using the View RNA FISH Assay kit (RiboBio) as previously described [18]. The images were obtained with an inverted fluorescence microscope (Nikon, Tokyo, Japan), and the ratio of nuclear/cytoplasm fluorescence intensity was calculated by ImageJ software (NIH, Bethesda, USA).

The GFP-LC3 plasmid was transfected into HepG2 cells for 24 h, followed by siRNA treatment, and then the cells were observed using a fluorescence microscope (Nikon). GFP-LC3 was also used for ApoB100 colocalization in autophagosomes when further incubated with apoB100 Cy3-labelled antibody and DAPI nuclear staining after 48 h of siRNA treatment.

### RNA pull-down and RNA immunoprecipitation assay

RNA pull-down was performed as previously described [19]. First, *lncRP11-675F6.3* and its antisense RNA were transcribed *in vitro* using the pGEM-3Z vector and T7 RNA polymerase RNA production system (Promega, Madison, USA). RNA was purified with an RNeasy® Mini kit (Qiagen, Valencia, USA) and treated with DNase I (Quanta Biosciences, Beverly, USA). RNAs were labelled with biotin using T4 RNA ligase sense, and the labelled RNA was captured with streptavidin magnetic beads. HepG2 cells were lysed and the extracts were mixed with the magnetic beads. The binding proteins on the beads were eluted and subsequently separated through SDS-PAGE followed by silver staining (Thermo Scientific, Waltham, USA). The differential band was cut and then subjected to be analyzed by Q Exactive LC-MS/MS (Thermo Scientific). The candidate binding protein was further verified by western blot following RNA pull-down.

RNA immunoprecipitation (RIP) was performed according to the manufacturer’s protocol (Millipore). Briefly, cells were lysed and immunoprecipitated using an antibody against HK1 (1:50; Millipore) with protein A/G magnetic beads. The complexes bound to the magnetic beads were immobilized with a magnet, and the beads were washed to remove unbound materials. Finally, binding RNA was extracted and analyzed by real-time fluorescence quantitative PCR (RT-qPCR).

### Lipid analysis

Both triglyceride and cholesterol levels in HepG2 cells and liver tissues were measured using their corresponding enzymatic assay kits (Jiancheng Biology Engineering Institute, Nanjing, China) according the manufacturer’s protocols, and the triglyceride (TG)/total cholesterol (TC) levels were normalized to the total protein concentration. VLDL level was determined using an ELISA kit (Jiancheng Biology Engineering Institute) according the manufacturer’s instructions.

Lipid droplets were prepared by Oil Red O staining (Sigma) as described previously [20]. The images were observed using an optical microscope (Nikon) and quantified by ImageJ software (NIH).

### Hematoxylin-eosin staining

The liver tissues were harvested and fixed in 4% formaldehyde overnight, dehydrated in 70% ethanol, cleared in xylene and embedded in paraffin. All liver tissues were then cut into 5 μm thick sections, dewaxed with xylene and stained with hematoxylin eosin (HE). After drying, sections were observed and photographed under an optical microscope (Nikon). The frozen sections were used for Oil Red O staining. Briefly, frozen sections were placed in 10% formalin and treated in propylene glycol. Then, the slides were placed in Oil Red O staining solution and heated to 60°C for 6 min, followed by treatment with modified Mayer’s hematoxylin and observation under a microscope.

### Real-time quantitative PCR (RT-qPCR)

Total RNA was extracted with Trizol and then reverse-transcribed into cDNA using the High-Capacity Reverse Transcription kit (Invitrogen). RT-qPCR was carried out using SYBR Green (Invitrogen) with a One Step Plus Real-Time PCR kit (Applied Biosystems, Foster City, USA). The relative RNA expression level was normalized to *β-actin* according to the 2 ^‒ΔΔCt^ calculation method. All primer sequences are shown in Supplementary Table S3.

Nuclear and cytoplasmic fractions were separated using the Cytoplasmic/Nuclear Purification kit (Norgen, Belmont, USA) according to the manufacturer’s instructions. The extract of each fraction was detected by RT-qPCR to quantify *lncRP11-675F6.3* separately using *U6* snRNA, *β-actin*, 18S rRNA and *GAPDH* as internal references.

### Western blot analysis

Total proteins were extracted from HepG2 cells and liver tissues with RIPA buffer (Cowin Biotech, Beijing, China). The protein concentrations were determined using a BCA-100 Protein Quantitative Analysis kit (Beyotime, Shanghai, China). Then, the proteins were subjected to SDS-PAGE and transferred to PVDF membranes (Millipore). The membranes were blocked and incubated with primary antibodies overnight at 4°C. Primary antibodies included monoclonal antibodies against ApoB100, ApoE, ApoC3, MTTP, LC3, and p62 (Abcam, Cambridge, USA), monoclonal antibodies against HK1 (Millipore), and antibody against β-actin (Beyotime). Then membranes were incubated with HRP-labelled secondary antibodies (Bio-Rad, Hercules, USA) for 1 h. Finally, the protein bands were visualized using ECL reagent (Bio-Rad).

### Statistical analysis

Data are represented as the Mean±Standard error. For the significance test, Student’s *t* test and ANOVA were used to determine significant differences between two groups and among three or more groups, respectively. A *P* value less than 0.05 was considered to be statistically significant.

## Results

### *LncRP11-675F6.3* is upregulated in response to rapamycin treatment

**Figure 1:**
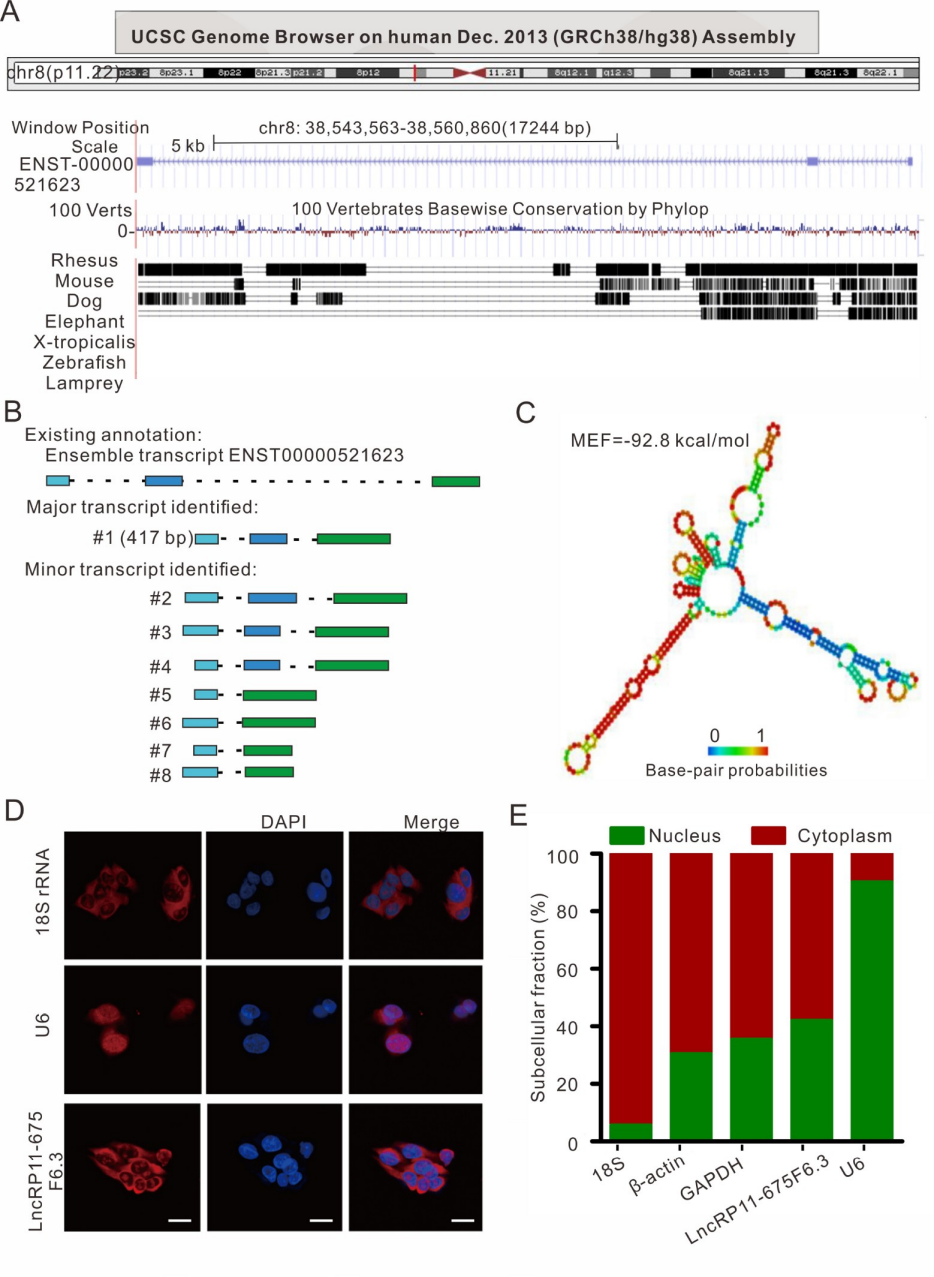
Figure 1 Characterization of *lncRP11-675F6.3* in the human genome and cellular distribution (A) Localization in the human genome and multiz alignment from the UCSC Genome Browser. (B) Alternative lncRP11-675F6.3 transcripts were identified by RLM-RACE. (C) Predicted secondary structure for lncRP11-675F6.3 using the RNA fold web server. (D) Subcellular localization of lncRP11-675F6.3 using RNA FISH and (E) ratio of cytoplasmic/nuclear using qPCR. GAPDH , β-actin and 18S rRNA were used as the internal controls for cytoplasmic RNA, and U6 for nuclear RNA.

To identify novel lncRNAs potentially involved in cellular metabolism, HepG2 cells were treated with rapamycin to inhibit the mTOR pathway. Total RNA was extracted and subjected to analysis using a lncRNA microarray ( Supplementary Figure S1). The results showed that there were 110 differentially expressed lncRNA transcripts, of which 85 were upregulated and 25 were downregulated with a fold change cut-off of ≥1.5 compared with the negative control ( Supplementary Table S4). A long intergenic noncoding RNA (probeName: p15666, gene ID: ENSG00000253361.1, gene_name: RP11-675F6.3, named *lncRP11-675F6.3* below) with unknown function was identified to be upregulated by 2.11- *fold*. It localized on human chromosome 8p11.22 (38, 543, 563-38, 560, 860) and was conserved by phyloP in mammals aligned to rhesus monkey, mouse, dog, and elephant ( Figure 1A). Eight transcripts were cloned using RLM-RACE, and the longest transcript was 417 nucleotides in full length, including 3 exons and 2 introns ( Figure 1B and Supplementary Table S5). The CPC score and the coding potential of *lncRP11-675F6.3* were 0.00224 and −1.15822, respectively ( Supplementary Table S6), which indicated the lack of coding potential. RNA secondary structure prediction showed that *lncRP11-675F6.3* had many peculiar stem-loop structures, which suggested its binding potential to DNA, RNA or proteins ( Figure 1C) [21].

### *LncRP11-675F6.3* mainly localizes in the cytoplasm and regulates triglyceride level in hepatocytes

**Figure 2:**
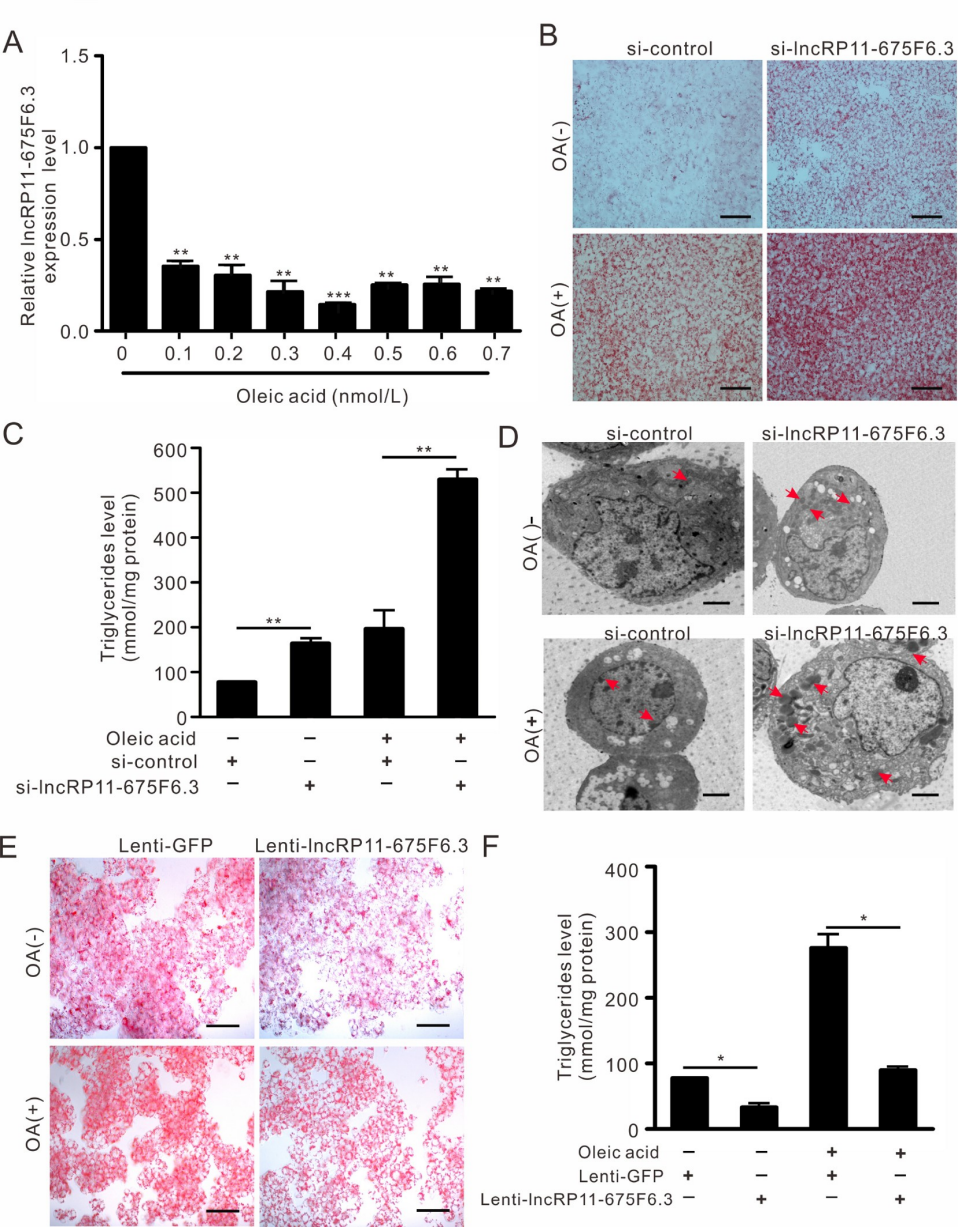
Figure 2 *LncRP11-675F6.3* regulates triglyceride level in HepG2 cells (A) LncRP11-675F6.3 expression in response to oleic acid at different concentrations for 24 h. (B) Oil Red O staining, (C) TG enzymatic assay, and (D) transmission electron microscopy (red arrows indicate the lipid droplets) showed significantly increased triglyceride contents in HepG2 cells compared with the control following siRNA treatment (10 μM) for 24 h. (E) Oil Red O staining and (F) TG enzymatic assay showed that lentivirus-mediated overexpression of lncRP11-675F6.3 suppressed triglyceride accumulation in HepG2 cells treated with oleic acid (0.4 mM) for 24 h. Data were analyzed by one-way ANOVA with Tukey’s post hoc test. *P<0.05, **P<0.01, *** P<0.001. Lenti-lncRP11-675F6.3 represents the stable lncRNA-overexpressing HepG2 cell lines; Lenti-GFP represents GFP-expressing HepG2 cell lines.

*LncRP11-675F6.3* was expressed in several types of hepatocytes, including HL7702 (LO2), Huh7 and HepG2 ( Supplementary Figure S2A). To determine the subcellular localization of *lncRP11-675F6.3* in hepatocytes, fluorescence *in situ* hybridization (FISH) and RT-qPCR experiments were performed, and the results showed that *lncRP11-675F6.3* was distributed both in the nucleus and cytoplasm in HepG2 cells, whereas it was mainly localized in the cytoplasm ( Figure 1D, E). As mTOR regulates lipid metabolism, we further investigated whether lncRNAs respond to fatty acid stimulation. As shown in Figure 2A, when HepG2 cells were exposed to gradient concentrations of oleic acid (OA), *lncRP11-675F6.3* expression was significantly declined at various concentrations with 24 h of treatment. Moreover, knockdown of *lncRP11-675F6*. *3* resulted in higher triglyceride level and increased lipid droplets either with or without OA treatment, as revealed by Oil Red O staining ( Figure 2B and Supplementary Figure S2C), triglyceride enzymatic assay ( Figure 2C) and transmission electron microscopy ( Figure 2D). As expected, lentivirus-mediated overexpression of *lncRP11-675F6.3* significantly reduced triglyceride accumulation in HepG2 cells ( Figure 2E, F and Supplementary Figure S2E, F).

### LncRP11-675F6.3 regulates autophagy in HepG2 cells

**Figure 3:**
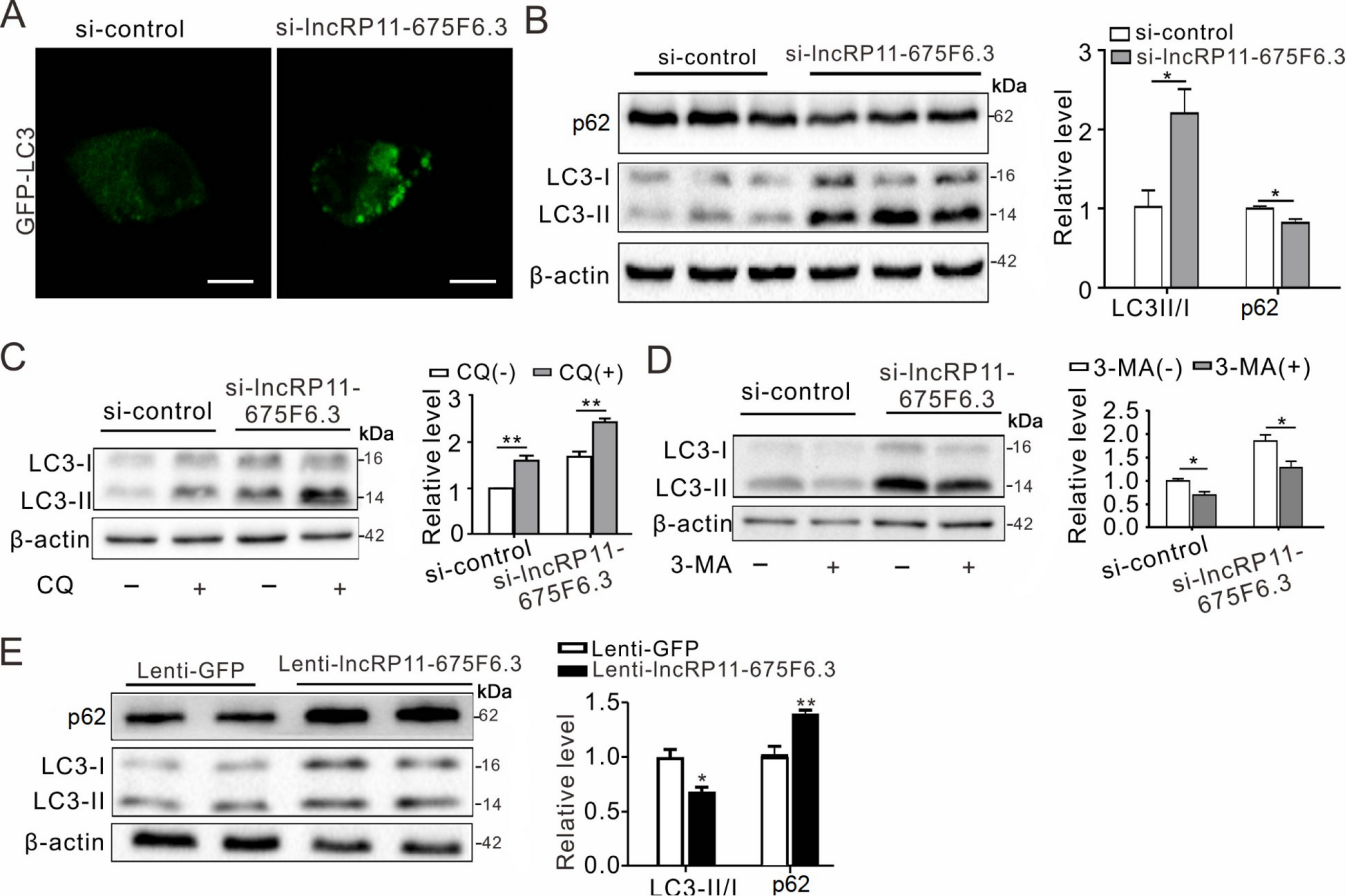
Figure 3 *LncRP11-675F6.3* regulates autophagy in HepG2 cells (A) GFP-LC3 fluorescence indicated enhanced autophagy in HepG2 cells after GFP-LC3 plasmid transient transfection for 24 h, followed by lncRP11-675F6.3 siRNA treatment. (B) Knockdown of lncRP11-675F6.3 induced autophagy in HepG2 cells with siRNA treatment (10 μM) for 24 h, represented by the amount of p62 and LC3II/I determined by western blot analysis. (C) HepG2 cells were treated with chloroquine (CQ, 4 μM) for 12 h and (D) 3-methyladenine (3-MA, 1 mM) for 12 h following knockdown of lncRP11-675F6.3. LC3II/I was evaluated by western blot analysis. (E) Lentivirus-mediated overexpression of lncRP11-675F6.3 repressed autophagy, as determined by western blot analysis. Data were analyzed by one-way ANOVA with Tukey’s post hoc test. * P<0.05, **P<0.01.

mTOR not only regulates lipid metabolism but also plays essential roles in autophagy. As *lncRP11-675F6.3* was elevated with mTOR inhibition, *lncRP11-675F6.3* was likely to be involved in regulating autophagy. The GFP-LC3 vector was transfected into HepG2 cells, and the fluorescence intensity was enhanced with knockdown of *lncRP11-675F6* . *3* ( Figure 3A). Furthermore, the LC3II/I ratio was significantly increased, and the p62 protein level was decreased, as determined by western blot analysis ( Figure 3B), which was verified through chloroquine (CQ) and 3-methyladenine (3-MA) autophagy inhibitor treatment ( Figure 3C, D). In contrast, overexpression of *lncRP11-675F6.3* in HepG2 cells led to a decreased autophagy tendency represented by the decreased ratio of LC3II/I and increased p62 protein expression ( Figure 3E).

### *LncRP11-675F6.3*-regulated VLDL protein levels are related to autophagy

**Figure 4:**
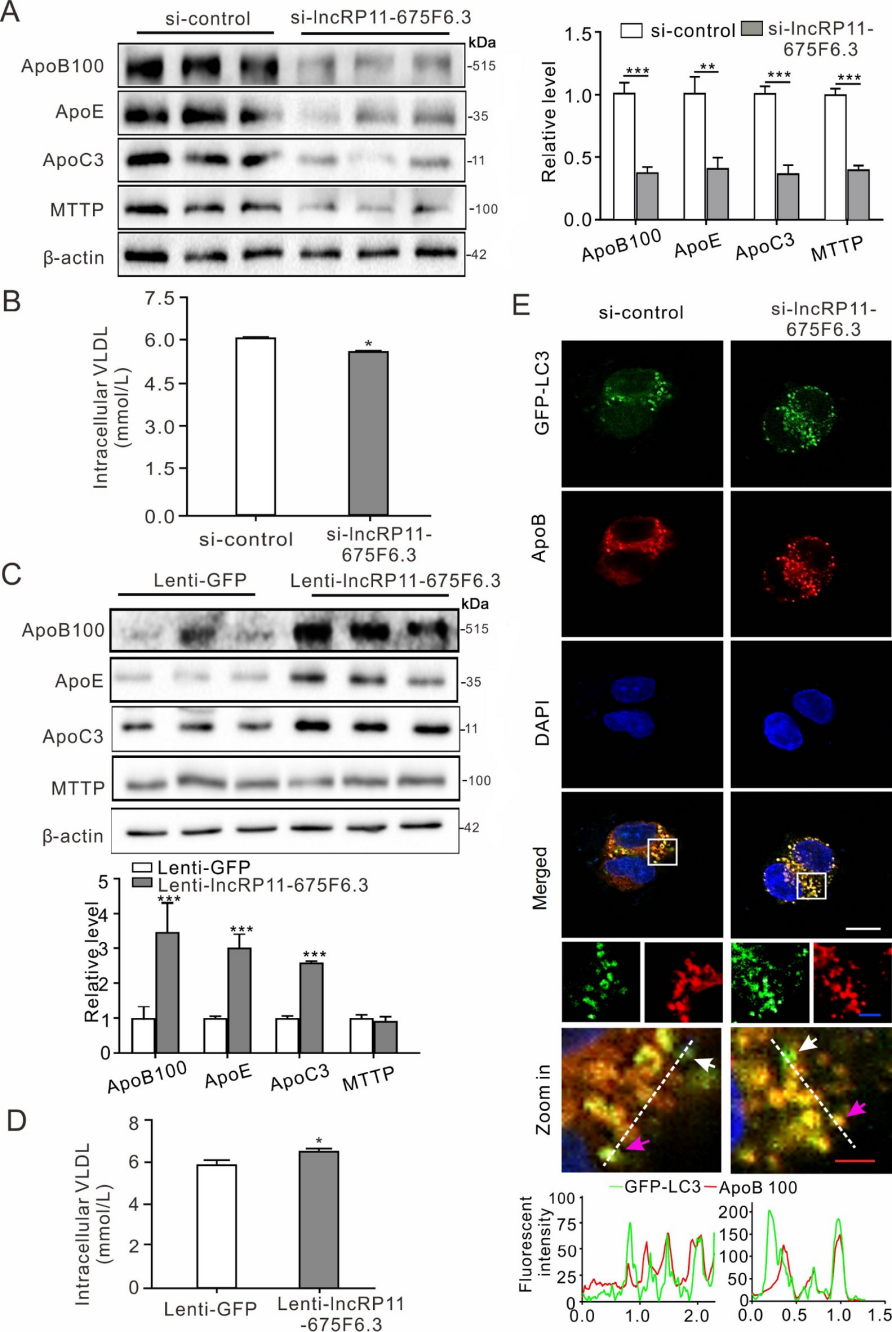
Figure 4 *LncRP11-675F6.3* regulates VLDL-related proteins and links ApoB100 to autophagy (A) Western blot analysis showed that the proteins related to VLDL, ApoB100, ApoE, MTTP, and Apoc3 were significantly reduced in HepG2 cells treated with siRNA (10 μM) for 48 h. (B) Intracellular VLDL was determined in HepG2 cells treated with siRNA (10 μM) for 48 h by ELISA. (C) Lentivirus-mediated overexpression of lncRP11-675F6.3 in HepG2 cells showed elevated levels of ApoB100, ApoE and Apoc3. (D) Intracellular VLDL was quantified by ELISA in lncRP11-675F6.3-overexpressing HepG2 cells. (E) Immunofluorescence microscopy showed the colocalization of LC3 (green) and ApoB100 (red) in HepG2 cells treated with siRNA (10 μM) for 48 h. Blue indicates DAPI. The zoom-in box showed the merged zone. White and pink arrows point to the LC3 dots (green) and merged (yellow), respectively. The bottom histogram indicates the fluorescence intensity of GFP-LC3 (green) and ApoB100 (red). Data were analyzed by one-way ANOVA with Tukey’s post hoc test. * P<0.05, **P<0.01, ***P<0.001.

To clarify how *lncRP11-675F6.3* links triglyceride regulation to autophagy, the expressions of genes involved in fatty acid synthesis, β-oxidation and VLDL assembly were detected by RT-qPCR, and the results showed that these genes related to triglyceride metabolism were significantly changed ( Supplementary Figure S3A). In particular, the VLDL proteins ApoB100, ApoC3, ApoE and MTTP were significantly reduced ( Figure 4A), which might lead to a decrease in intracellular VLDL levels ( Figure 4B). In the case of overexpression of lncRNA, the results were reversed, i.e., the above VLDL proteins as well as intracellular VLDL content were increased significantly ( Figure 4C, D and Supplementary Figure S3B). More interestingly, ApoB100 colocalized in autophagosomes with GFP-LC3, and the fluorescence intensity was enhanced with knockdown of *lncRP11-675F6*. *3* ( Figure 4E).

### *LncRP11-675F6.3* interacts with hexokinase-1

**Figure 5:**
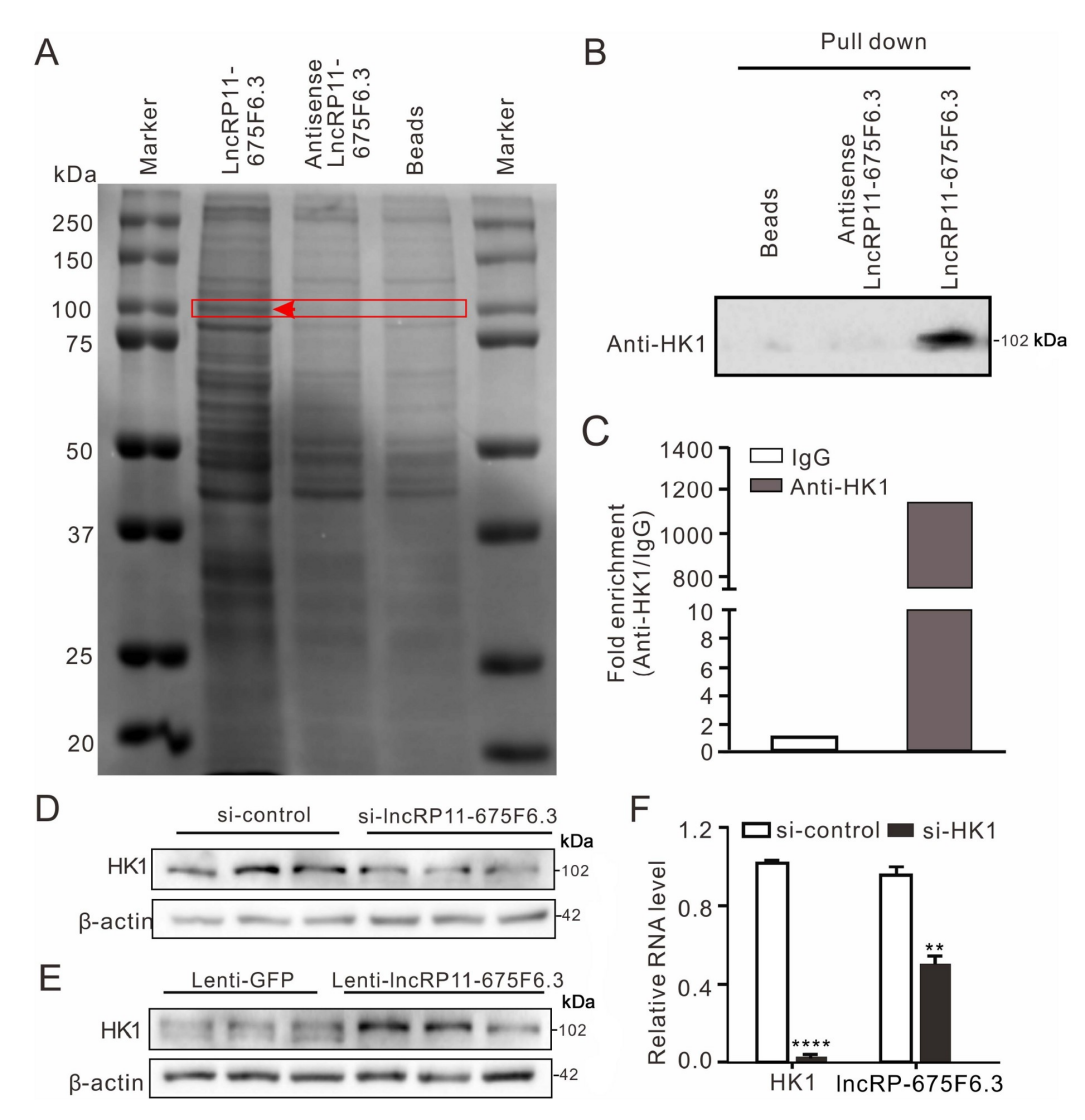
Figure 5 *LncRP11-675F6.3* binds to HK1 (A) RNA pull-down assay showed that one band (~100 kDa) existed only in lncRP11-675F6.3 precipitates. HK1 was identified by LC-MS/MS. The red arrow indicates the additional band. (B) Western blot analysis confirmed the binding of HK1 with lncRP11-675F6.3 following RNA pulldown. (C) RIP assay showed that HK1 interacts with lncRP11-675F6.3 with high affinity. (D) Knockdown or (E) overexpression of lncRP11-675F6.3 resulted in the downregulation or upregulation of HK1 as determined by western blot analysis. (F) Knockdown of HK1 in HepG2 cells led to downregulation of lncRP11-675F6.3 as revealed by RT-qPCR. Data were analyzed by one-way ANOVA with Tukey’s post hoc test. **P<0.01, ****P<0.0001.

Because lncRNAs generally interact with proteins to regulate gene expression, RNA pulldown was used to capture the proteins interacting with *LncRP11-675F6.3*. One differential band compared with an antisense control and beads was present in the silver staining at a position of 100 kDa in the gel after SDS-PAGE, from which the precipitates were separated by biotin-labelled *lncRP11-675F6.3* ( Figure 5A). Proteins in the specific band were identified by mass spectrometry ( Supplementary Table S7), one of which was hexokinase-1 (HK1), a protein related to glucose and autophagy [22]. Western blot analysis was used to detect RNA pulldown precipitates using a specific antibody against HK1 ( Figure 5B). The specific interaction between HK1 and *lncRP11-675F6.3* was further confirmed by RNA immunoprecipitation (RIP) with an HK1 antibody ( Figure 5C). *LncRP11-675F6.3* bound to HK1 was quantified by RT-qPCR. *LncRP11-675F6.3* was significantly enriched in anti-HK1 precipitates compared with the IgG control. Moreover, knockdown of *lncRP11-675F6*. *3* induced a decrease of HK1 protein, and overexpression of *lncRP11-675F6.3* led to an increase of HK1 ( Figure 5D, E). Vice versa, knockdown of *HK1* resulted in significant downregulation of *lncRP11-675F6.3* expression ( Figure 5F).

### HK1 regulates triglycerides, VLDL and autophagy similarly to *lncRP11-675F6.3*

**Figure 6:**
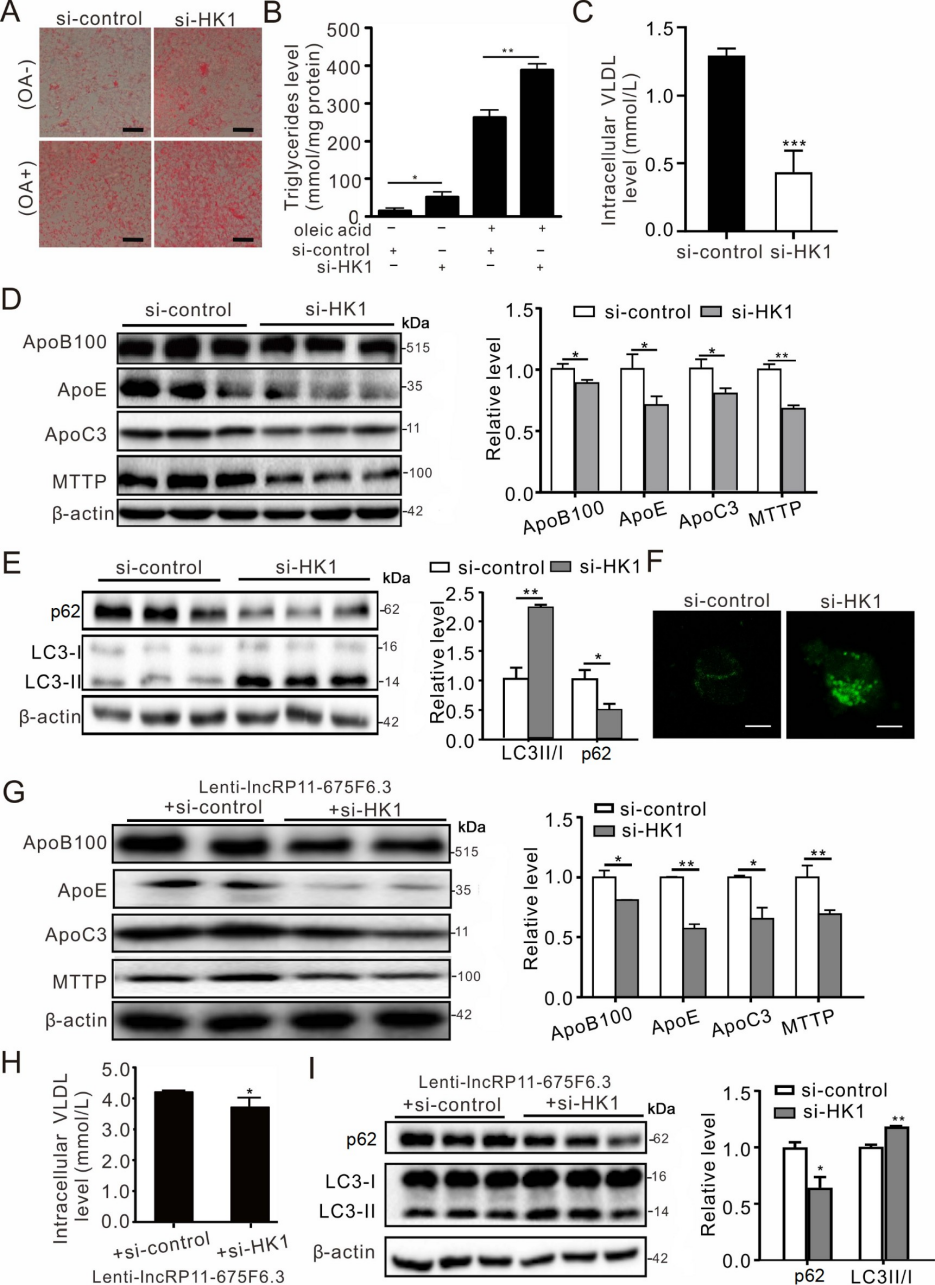
Figure 6 HK1 regulates triglyceride level and autophagy and is required for *lncRP11-675F6.3* (A) Oil Red O staining showed that triglyceride accumulation was significantly increased in HepG2 cells treated with HK1 siRNA for 48 h. (B) TG enzymatic assay indicated that triglyceride level was elevated with HK1 knockdown. (C) ELISA indicated that the levels of intracellular VLDL were decreased in HepG2 cells treated with HK1 siRNA. (D) Western blot analysis showed that ApoB100, ApoE, ApoC3 and MTTP were reduced with HK1 knockdown. (E) Western blot analysis showed reduced p62 and increased LC3II/I with HK1 knockdown. (F) GFP-LC3 fluorescence was enhanced with HK1 knockdown. (G) Western blot analysis indicated that ApoB100, ApoE, ApoC3 and MTTP were reduced with HK1 knockdown in lenti-lncRP11-675F6.3 HepG2 cells. (H) ELISA indicated that intracellular VLDL levels were reduced with HK1 knockdown in lncRP11-675F6.3-overexpressing HepG2 cells. (I) Western blot analysis showed p62 reduction and LC3II/I elevation with HK1 knockdown in lncRP11-675F6.3-overexpressing HepG2 cells. Data were analyzed by one-way ANOVA with Tukey’s post hoc test. * P<0.05, **P<0.01, ***P <0.001.

To test the effect of HK1 on triglyceride metabolism, siRNAs targeting HK1 were designed to inhibit HK1 expression in HepG2 cells ( Supplementary Figure S4A, B). Similar to *lncRP11-675F6.3*, HK1 also affected triglyceride level, VLDL proteins and autophagy in HepG2 cells. Knockdown of *HK1* resulted in significantly elevated triglyceride level, as revealed by Oil Red O staining ( Figure 6A) and TG enzymatic assay ( Figure 6B), as well as decreased VLDL level detected by ELISA ( Figure 6C). It was also found that the amounts of VLDL proteins ApoB100, ApoE, ApoC3 and MTTP were notably reduced ( Figure 6D), even with overexpression of *lncRP11-675F6.3* in HepG2 cells. These proteins also showed a decline in the same way ( Figure 6G, H), which indicated that *lncRP11-675F6.3* is most likely dependent on HK1 in regulating triglycerides. Furthermore, knockdown of *HK1* also led to the activation of autophagy, indicated by a reduction in p62 and an increased ratio of LC3II/I compared with controls ( Figure 6E, F, I).

### *lncRP11-675F6.3* and HK1 alleviate hepatic steatosis *in vivo*

**Figure 7:**
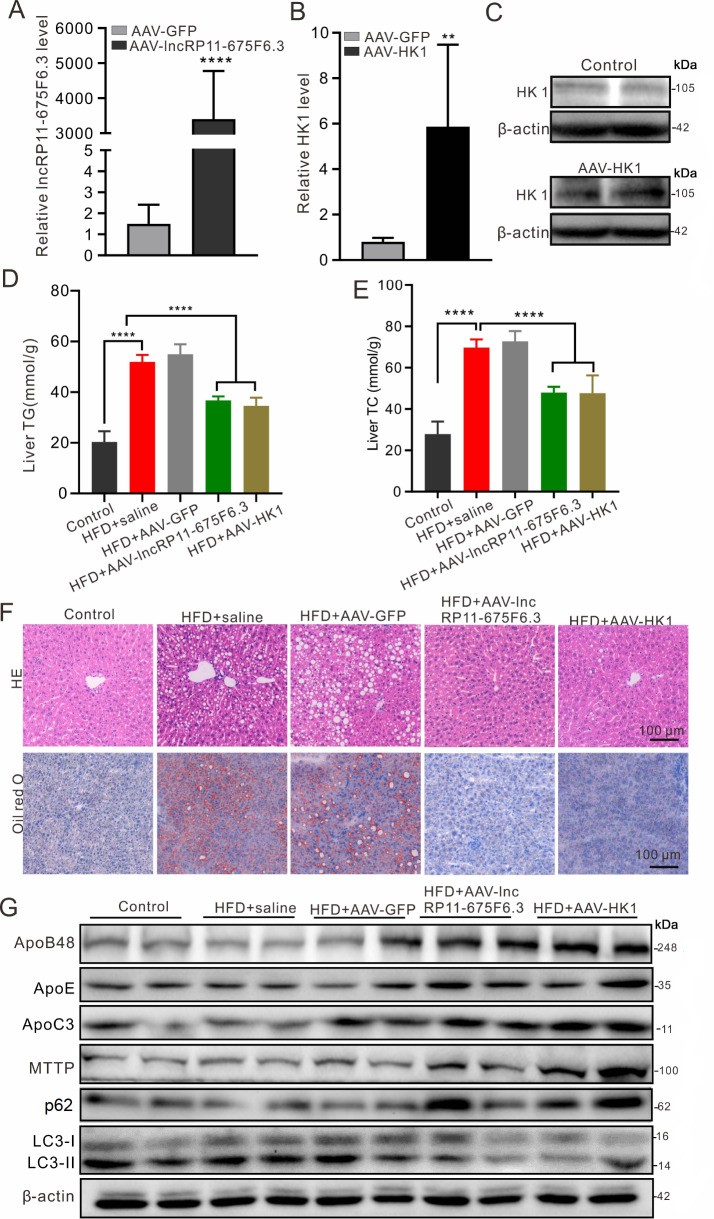
Figure 7 *lncRP11-675F6.3* and HK1 alleviate hepatic steatosis *in vivo* (A) LncRP11-675F6.3 and (B) HK1 RNA levels were significantly increased in livers with AAV-mediated lncRP11-675F6.3 and HK1 delivery. (C) HK1 expression was confirmed by western blot analysis. (D) TG and (E) TC levels in livers were detected using enzymatic assay kits and showed a reduction in both TG and TC in mice with AAV-mediated lncRP11-675F6.3 and HK1 delivery. (F) HE staining (upper) and oil red O staining (below) showed alleviated hepatic steatosis in mice with AAV-mediated lncRP11-675F6.3 and HK1 delivery. (G) Western blot analysis showed elevated expressions of ApoB48, ApoE, MTTP and Apoc3, increased p62 and decreased LC3II/I in mice with AAV-mediated lncRP11-675F6.3 and HK1 delivery. Data were analyzed by one-way ANOVA with Tukey’s post hoc test. **P<0.01, ****P<0.0001.

To confirm the roles of *lncRP11-675F6.3* and HK1 in regulating lipid metabolism *in vivo*, a fatty liver mouse model was established with HFD feeding for 9 weeks. With HFD feeding, all the mice showed continuous body weight elevation compared with the chow-fed group ( Supplementary Figure S5A). AAV-lncRP11-675F6.3 and AAV-HK1 were delivered via the tail vein each week for 9 consecutive times. After the mice were sacrificed, the expression of both *lncRP11-675F6.3* ( Figure 7A) and HK1 mRNA and protein ( Figure 7B, C) were increased in the liver tissues ( Supplementary Figure S5B) compared with other groups, whereas the levels of liver cholesterol and triglycerides were significantly reduced ( Figure 7D, E) in comparison with either the HFD group or the HFD AAV-GFP group. More significantly, hepatic steatosis was remarkably alleviated via HE and Oil Red O staining in both the AAV-lncRP11-675F6.3- and AAV-HK1-treated groups ( Figure 7F). Consistent with the results *in vitro*, mouse VLDL proteins for ApoB48, ApoE, ApoC3 and MTTP were significantly increased in liver tissue, whereas autophagy tended to be reduced, as revealed by elevation of p62 and decreased ratio of LC3II/I in the AAV-lncRP11-675F6.3- and AAV-HK1-treated groups ( Figure 7G).

## Discussion

In the present study, we identified one novel lncRNA named *lncRP11-675F6.3* that could regulate hepatocyte lipid metabolism. The majority of lncRNAs remain functionally unclear. Previous functional predictions of long noncoding RNAs were mainly based on gene coexpression data [23]. As lncRNAs can respond to exogenous signals and map to a specific signaling pathway [ 24, 25], they facilitate the functional determination of novel lncRNAs. For instance, mouse hepatic *LeXis* expression was induced in response to either a western diet or to LXR activation. Upregulation or downregulation of LeXis in hepatocytes affected the expressions of genes involved in cholesterol biosynthesis as well as the cholesterol levels in the plasma and liver [10]. According to this strategy, we herein used rapamycin to treat HepG2 cells with the expectation of identifying lncRNAs associated with the mTOR signaling pathway. A total of 110 differentially expressed lncRNA transcripts were found in our study and are thought to be involved in the downstream regulation of the mTOR signaling pathway.

mTOR regulates cell growth, protein synthesis, autophagy and lipid synthesis [26]. Therefore, we first observed the role of *lncRP11-675F6.3* in lipid metabolism in HepG2 cells in accordance with our research interests. When *lncRP11-675F6*. *3* was knocked down, triglyceride accumulation was significantly increased, as well as related genes such as *SREBP1c* *FASN*, and *ACSCL*, whereas *lncRP11-675F6.3* overexpression resulted in the downregulation of *FASN* and upregulation of *CPT1*. These results indicated that the upregulation of *lncRP11-675F6.3* in response to rapamycin treatment potentially facilitated the switch-on of catabolism, which was consistent with the subsequent effect of mTOR inhibition. In accordance with this conclusion, upregulation of *lncRP11-675F6.3* theoretically induced cell autophagy. However, knockdown of *lncRP11-675F6*. *3* unexpectedly led to autophagy. Therefore, *lncRP11-675F6.3* showed divergent roles downstream of the mTOR signaling pathway.

We subsequently investigated the link between triglyceride accumulation and autophagy in HepG2 cells when *lncRP11-675F6*. *3* was knocked down. As the amounts of VLDL assembly related proteins, such as ApoB100, MTTP, ApoC3 and ApoE, were significantly reduced with *lncRP11-675F6*. *3* knockdown and accompanied by autophagy, we predicted that autophagy might mediate the degradation of VLDL resulted from *lncRP11-675F6.3* suppression. It was previously reported that autophagy was involved in the Sortilin-mediated degradation of ApoB100 [27]; therefore, we detected the colocalization of ApoB100 in autophagosomes. Finally, the results revealed that *lncRP11-675F6.3* linked autophagy to VLDL assembly, which indicated a potential target for modulating lipid metabolism.

LncRNAs generally interact with proteins and microRNAs, acting as signals, decoys, guides, and scaffolds, to modulate gene expression [28]. Therefore, we further identified the binding proteins of *lncRP11-675F6.3* by mass spectrometry, and approximately 20 proteins were identified at nearly 100 kDa, one of which was HK1, an enzyme encoded by the *HK1* gene, which exists on the outer membrane of mitochondria and is responsible for the first obligatory step of glucose metabolism [29]. HK1 was reported recently, and higher p-AKT level activated by *LINC00470* could inhibit ubiquitination of HK1, which affected glycolysis and inhibited cell autophagy [22]. Therefore, we selected HK1 for further validation. As the results showed, lowering or raising HK1 levels had a consistent effect on triglycerides and autophagy with *lncRP11-675F6.3.* Moreover, it was suggested that *lncRP11-675F6.3* might depend on HK1 in lipid metabolism. Although the role of HK1 in lipid metabolism and autophagy has not been completely demonstrated, it is likely that *lncRP11-675F6.3* interacts with HK1, potentially connecting glucose metabolism to lipid metabolism.

Although the detailed molecular mechanism remains unknown, we tested the effects of *lncRP11-675F6.3* and HK1 *in vivo* and found that either *lncRP11-675F6.3* or HK1 could significantly relieve HFD-induced NAFLD by inhibiting autophagy and promoting VLDL assembly. Herein, our study revealed the novel function of *lncRP11-675F6.3* and HK1, suggesting a potential lipid regulatory pathway and therapeutic strategy for NAFLD.

In summary, the present study revealed the primary function of one novel long noncoding RNA, *lncRP11-675F6.3*, indicating its potential role downstream of the mTOR pathway and in the lipid metabolism regulatory network. Moreover, the interaction between *lncRP11-675F6.3* and HK1 may provide a new approach for fatty liver treatment.

## Supporting information

Supplementary_information-ABBS

## Supplementary Data

Supplementary data is available at *Acta Biochimica et Biophysica Sinica* online.
