# Supplementary material for: LncRP11-675F6.3 responds to rapamycin treatment and reduces triglyceride accumulation via interacting with HK1 in hepatocytes by regulating autophagy and VLDL-related proteins: LncRNA with HK1 regulates triglycerides, autophagy and VLDL
Source: Acta Biochim Biophys Sin (Shanghai). 2023 May 24;55(10):1606–17. doi: 10.3724/abbs.2023091 (PMC10577451; doi:10.3724/abbs.2023091)
Supplement: Supplementary_information-ABBS [file Supplementary_information-ABBS.pdf]

SUPPORTMENTAL INFORMATION:

LncRP11-675F6.3 interacting with HK1, responses to rapamycin and reduces triglycerides in hepatocytes via regulating autophagy and VLDL

Ling-Ling Wang[1,*], Xiao-Juan Fang[1,*], Zi-You Yang[1], Xue-Ling Li[1], Meng-Di Cheng[1], Liang Cheng[1], Gang-Lin Wang[1], Wei Li[1, §] and Lin Liu[1,2, §]

[1] Key Laboratory of Laboratory Medicine, Ministry of Education of China, Zhejiang Provincial Key Laboratory of Medical Genetics, School of Laboratory Medicine and Life Sciences, Wenzhou Medical University, Wenzhou, 325035, China

[2] Zhuji Affiliated Hospital of Wenzhou Medical University, Shaoxing, 311800, China.

Table S1. **SiRNA oligos sequences**

Target gene	Number	Sequences
lncRP11-675F6.3	siRNA-1	GCUACCAUUUCCCUGGUCUTT
	siRNA-2	CCGUGUAGAUGAAGACAAATT
	siRNA-3	GCAAGAAGUACACUUCCAUTT
HK1	siRNA-1	CCUGGACAAAGCGAUUUAATT
	siRNA-2	CCAGUGAUGUGUCAGCCAUTT
	siRNA-3	GCCUUUGGAGACGAUGGAUTT
	si-NC	UUCUCCGAACGUGUCACGU

Table S2. **Primers for experiments in this study**

Primer Description	Primer sequence (5’-3’)
Sequences of gene–specific PCR primers used for RACE	
3’RACE outer gsp Primer	ACTAGTTCTGACCGGAGACCAGGGA
3’RACE inner gsp Primer	TCCCTGGTCTCCGGTCAGAACTAGT
5’RACE outer gsp Primer	GACCTGAGGGTTTCCCATTT
5’RACE inner gsp Primer	TGTTTCTCTGCCATCTCCTAAAG
Sequences of the nucleotide sequence of full-length LncRP11-675F6.3 PCR primers.	
Forward1	TTTTTTCATTCCAGGCTGTGGATCC
Forward2	ATTCCAGGCTGTGGATCCGGTCG
Forward3	TTCCAGGCTGTGGATCCGGTCGTG
Reverse1	TGGTTGTTTAAATAAGATGGAAGC
Reverse2	GTGCTTAGGAGGTTTTGATATTA
Sequences of primers used for pCDH-EF1-MCS-T2A-Puro-LncRP11-675F6.3/Antisense-LncRP11-675F6.3 construction	
Forward	GCGTCGACTTCCAGGCTGTGGATCCG
Reverse	GCGTCGACGTGCTTAGGAGGTTTTGA

Table S3. **Primers used for real time quantitative PCR**

Gene name	Forward primer (5'→3')	Reverse primer (5'→3')
LncRP11-675F6.3	ATCGCCGTGTAGATGAAGAC	GGCAATTCTGCTTGGTGTAG
GAPDH	AGCTCACTGGCATGGCCTTC	CGCCTGCTTCACCACCTTCT
18S	GTAACCCGTTGAACCCCATT	CCATCCAATCGGTAGTAGCG
Homo-β-actin	GGCACCACACCTTCTACAAT	GCCTGGATAGCAACGTACAT
HK1	AGCGAGGGGACTATGATGCCAA	GTGCCAGTGCCGATGATCAGGC
FAS	TACGACTACGGCCCTCATTT	CCATGAAGCTCACCCAGTTATC
CIDEB	GACACAGTCTCACCTCTCATTT	CACCTACTGGAGAAGCCATAAG
SREBP-1C	GGGACAAGGAATTCTCGGATG	GAACTGATGGAGAAGCTGTAGG
PPARγ	CTCAAACGAGAGTCAGCCTTTA	GTGGGAGTGGTCTTCCATTAC
ACC	GAGGTGGATCGGAGATTTCATAG	AGGCTCCAGATGACGATAGA
PGC-1	TGAACTGAGGGACAGTGATTTC	CCCAAGGGTAGCTCAGTTTATC
ApoC3	AGCCTTGACCTTTCACATC	AAGTCAAACCCTGCCATCTC
MTTP	AGAGTAGCTGAGGGAGGATTAG	GAACAGCTGTGGAAAGATTTGG
ApoE	GAAGACCAGGAGGGAGTTAAAT	TTCCAAGCCTTGTTGCATTATC
ApoB100	GCAGGAGCCAGGTCAATA	CCCGCTGAGCTACATGAAT
ACLY	GCCCATCCCCAACCAGCCAC	TTGCAGGCGCCACCTCATCG
SCD1	ACGACATTCGCCCTGATATAA	CCCAAGTGTAGCAGAGACATAAG
PPARα	CCCAAAGCAGAAAGCAGAAAC	CCCAGAGGGTGTAACCAATATG
CPT-1	TCCTGGTGGGCTACAAATTAC	ACAGCAGATCCATGGCATAATA
ACSL3	GACCTCCGAAACACTTCCATAC	AGACTGAGCCACCACAATTAC
ACSL4	CCCTCCTTCTGTTGTACCTAATG	CCCTATGCTGCTCTTCTCAAA
Hus-β-actin	CGACAATGGTACCGGCTTTG	TCAACGTGTAAATCCTTAACTTC

Table S4. **Differential expression (fold change≥ |1.5|) of lncRNAs in HepG2 cells with rapamycin treated compared with DMSO treated cells using microarray assay**

Probe Name	Fold change	lncRNA ID	Probe Name	Fold change	lncRNA ID
p1448	1.5820193	ENST00000421878.1	p37311_v4	1.5641204	ENST00000436616.2
p26639	1.6401258	HIT000061665	p1061	1.5075101	ENST00000412628.1
p33968_v4	1.5798925	ENST00000457025.1	p15104	1.5325483	ENST00000418546.1
p18258	1.5572791	TCONS_00019561	p15666	2.115597	ENST00000521623.1
p29943	1.5660087	TCONS_00011776	p13660	1.5347507	ENST00000451894.2
p15384	1.520313	ENST00000452320.1	p476	1.5609152	ENST00000448869.1
p13921	2.0992937	ENST00000510311.1	p35600_v4	1.5869076	TCONS_00018797
p43270_v4	1.6680751	XR_429785.1	p11342	1.7624352	ENST00000441531.1
p619	1.6286991	ENST00000567538.1	p29642	1.5517131	TCONS_00020390
p7811	1.706079	ENST00000580048.1	p1563	1.9830228	ENST00000431956.1
p1549	1.6716844	ENST00000441257.1	p20502	1.5267211	TCONS_00026273
p36827_v4	1.6319674	ENST00000606790.1	p28084	1.5995122	nc-HOXD3-37
p3752	1.6410336	ENST00000550049.1	p34724_v4	1.5954752	ENST00000598560.1
p7857	2.073026	ENST00000572856.1	p40812_v4	1.5347703	NR_105045.1
p1714	1.5752276	ENST00000452391.1	p43713_v4	1.5292274	NR_038339.2
p23349	1.6093154	TCONS_00012390	p20248	1.5030806	TCONS_00025807
p20883	1.5625278	TCONS_00005195	p25330	1.5352379	NR_037665.1
p28085	1.5180674	nc-HOXD3-38	p18259	2.288485	TCONS_00019562
p23949	2.2379682	TCONS_00014978	p24415	1.6366384	TCONS_00016543
p20526	1.5268383	TCONS_00026302	p37209_v4	1.5113044	ENST00000610144.1
p95554	1.5588073	RNS_636_144	p12792	1.5105075	ENST00000508021.1
p15938	1.5466051	ENST00000500112.1	p30297	1.6116668	ASO2000
p147005	1.578985	p0109_imsncRNA184	p15381	1.6520046	ENST00000429408.1
p12847	1.5402753	ENST00000437514.1	p8840	1.5883548	ENST00000422045.1
p5054	1.5595888	ENST00000563044.1	p23145	1.5034858	TCONS_00012039

p14260	1.5728339	ENST00000454981.1	p6838	1.6218436	ENST00000590740.1
p20974	1.6455914	TCONS_00005314	p22979	1.7063667	TCONS_00009975
p34837_v4	1.5729685	ENST00000454488.1	p859	1.9045222	ENST00000455431.1
p33500	1.6097283	ENST00000424536.1	p15422	1.5193408	ENST00000420058.1
p15667	2.1257782	ENST00000519764.1	p28554	1.5517513	ASO2205
p5928	1.5172534	ENST00000565498.1	p19130	1.5044632	TCONS_00022125
p37870_v4	1.5141461	ENST00000607718.1	p5881	1.5744731	ENST00000572706.1
p17784	1.5073096	TCONS_00000054	p28922	1.8870001	AK024556.1
p40143_v4	1.5836046	XR_427390.1	p28602	1.5466589	ASO1967
p18827	1.5553504	TCONS_00020392	p25087	1.8039649	XR_109464.1
p3178	1.5019423	ENST00000546686.1	p2795	1.5804647	ENST00000499732.1
p9475	1.6129043	ENST00000421437.1	p34784_v4	1.7223027	ENST00000449569.1
p26640	1.5609372	HIT000220650	p18826	1.5955825	TCONS_00020388
p33486	1.6072384	ENST00000592276.1	p33898	1.5549219	HIT000325986
p28641	1.9359219	ASO2287	p7854	1.9982295	ENST00000574411.1
p7855	2.0963182	ENST00000573355.1	p7856	1.7278254	ENST00000573177.1
p35353_v4	1.6351771	ENST00000422093.1	p4091	1.6244485	ENST00000434117.1
p1174	1.5249922	ENST00000440377.1			
p28933	1.5882448	ENST00000461448.1	p7291	1.5665663	ENST00000417404.1
RNA33518	1.5659382	scaRNA_114_130	p17876	1.6102641	TCONS_00002269
p12707	1.5680058	ENST00000441504.1	p126	1.6272087	ENST00000474814.1
p12096	1.5657703	ENST00000434309.1	p24591	1.5680313	TCONS_00030038
p28919	1.7478207	NR_004435.1	p23454	1.5578092	TCONS_00011825
p1630	2.3147109	ENST00000491934.2	p14658	1.5083133	ENST00000532353.1
p4537	1.51722	ENST00000500036.2	p28934	1.5323317	ENST00000488745.1
p26871	1.6438167	HIT000383087	p38733_v4	1.5046706	ENST00000609990.1
p28920	1.822818	EU035784.1	p36841_v4	1.5438207	ENST00000384581.1
RNA147698	1.6241001	p0802_imsncRNA596	p39997_v4	1.5457513	XR_427233.1
p6246	1.647643	ENST00000567888.1	RNA147023	1.50505	p0127_imsncRNA205
p701	1.5290793	ENST00000458044.1	p18393	1.5644987	TCONS_00019803

p9006	1.6196374	ENST00000425678.1			

Note: black indicated upregulated; red indicated downregulated.

Table S5. **Eight transcripts sequence of lncRP11-675F6.3**

Transcripts	Sequences
Transcript 1:	TTCCAGGCTGTGGATCCGGTCGTGGGAAGCAGGGCCGCACACAT
227nt	ACAGAGCTGAACTTGTATCGCCGTGTAGATGAAGACAAACACCT
	TTAGGAGATGGCCGAGAAACAAAATGGGAAACCCTCAGGTCTCC
	AAATGACTACACCAAGCAGAATTGCCCCACCAGCCTGCACCATTC
	ATTTCTAGACTATGAGCAAGAAGTACACTTCCATCTTATTTAAACA
	ACCA
Transcript 2:	ATTAGGCTGTGGATCCGGTCGTGGGAAGCAGGGCCGCACACATA
344nt	CAGAGCTGAACTTGTATCGCCGTGTAGATGAAGACAAACACCTTT
	AGGAGATGGCCGAGAAACAAAATGGGAAACCCTCAGGTCTCCA
	AATGACTACACCAAGCAGAATTGCCCCACCAGCCTGCACCATTCA
	TTTCTAGACTATGAGCAAGAAGTACACTTCCATCTTATTTAAACAA
	CCATATTTGGGGTTTTTGTTGTTGTTATTGTTGTTGTTATAGGAGCT
	TAGCTTTTACTCTAAGAAATACAGCTACTCAACTCACTTCATTAGG
	ATCTTAACTATAATATCAAAACCTCCTAAGCAC
Transcript 3:	TTCCAGGCTGTGGATCCGGTCGTGGGAAGCAGGCTGGTCTCCAA
294nt	CTTCTGGCTGCAGGTGATCCTCCCTCCTTGGCCTGGCAAAGTGCT
	GGGATTACAGGGCCGCACACATACAGAGCTGAACTTGTATCGCCG
	TGTAGATGAAGACAAACACCTTTAGGAGATGGCCGAGAAACAAA
	ATGGGAAACCCTCAGGTCTCCAAATGACTACACCAAGCAGAATT
	GCCCCACCAGCCTGCACCATTCATTTCTAGACTATGAGCAAGAAG
	TACACTTCCATCTTATTTAAACAACCA
Transcript 4:	ATTCCAGGCTGTGGATCCGGTCGTGGGAAGCAGGGCCGCACACA
351nt	TACAGAGCTGAACTTGTATCGCCGTGTAGATGAAGACAAACACCT
	TTAGGAGATGGCCGAGAAACAAAATGGGAAACCCTCAGGTCTCC
	AAATGACTACACCAAGCAGAATTGCCCCACCAGCCTGCACCATTC
	ATTTCTAGACTATGAGCAAGAAGTACACTTCCATCTTATTTAAACA
	ACCATATTTGGGGTTTTTGTTGTTGTTATTGTTGTTGTTATAGGAGC
	TTAGCTTTTACTCTAAGAAATACAGCTACTCAACTCGCTTCATTAG
	GATCTTAACTATAATATCAAAACCTCCTAAGCAC
Transcript 5:	TTTTTTCATTCCAGGCTGTGGATCCGGTCGTGGGAAGCGGGGCCG
235nt	CACACATACAGAGCTGAACTTGTATCGCCGTGTAGATGAAGACAA

	ACACCTTTAGGAGATGGCCGAGAAACAAAATGGGAAACCCTCAG<br>GTCTCCAAATGGCTACACCAAGCAGAATTGCCCCACCAGCCTGC<br>ACCATTCATTTCTAGACTATGAGCAAGAAGTACACTTCCATCTTAT<br>TTAAACAACCA
Transcript 6:<br>302nt	TTCATTCCAGGCTGTGGATCCGGTCGTGGGAAGCAGGCTGGTCTC<br>CAACTTCTGGCTGCAAGTGATCCTCCCTCCTTGGCCTGGCAAAGT<br>GCTGGGATTACAGGGCCGCACACATACAGAGCTGAACTTGTATCG<br>CCGTGTAGATGAAGACAAACACCTTTAGGAGATGGCCGAGAAAC<br>AAAATGGGAAACCCTCAGGTCTCCAAATGACTACACCAAGCAGA<br>ATTGCCCCACCAGCCTGCACCATTCATTTCTAGACTATGAGCAAG<br>AAGTACACTTCCATCTTATTTAAACAACCA
Transcript 7:<br>324nt	TTCCAGGCTGTGGATCCGGTCGTGGGAAGCAGTAAAGATGGGAT<br>CTTGTTCTGTTGCCCAGGCTGGTCTCCAACTTCTGGCTGCAAGTG<br>ATCCTCCCTCCTTGGCCTGGCAAAGTGCTGGGATTACAGGGCCGC<br>ACACATACAGAGCTGAACTTGTATCGCCGTGTAGATGAAGACAA<br>ACACCTTTAGGAGATGGCCGAGAAACAAAATGGGAAACCCTCAG<br>GTCTCCAAATGACTACACCAAGCAGAATTGCCCCACCAGCCTGC<br>ACCATTCATTTCTAGACTATGAGCAAGAAGTACACTTCCATCTTAT<br>TTAAACAACCA
Transcript 8:<br>417nt	TTCCAGGCTGTGGATCCGGTCGTGGGAAGCAGGCTGGTCTCCAA<br>CTTCTGGCTGCAAGTGATCCTCCCTCCTTGGCCTGGCAAAGTGCT<br>GGGATTACAGGGCCGCACACATACAGAGCTGAACTTGTATCGCCG<br>TGTAGATGAAAACAAACACCTTTAGGAGATGGCCGAGAAACAAA<br>ATGGGAAACCCTCAGGTCTCCAAATGACTACACCAAGCAGAATT<br>GCCCCACCAGCCTGCACCATTCATTTCTAGACTATGAGCAAGAAG<br>TACACTTCCATCTTATTTAAACAACCATATTTGGGGTTTTTGTTGTT<br>GTTATTGTTGTTGTTATAGGAGCTTAGCTTTTACTCTAAGAAATAC<br>AGCTACTCAACTCACTTCATTAGGATCTTAACTATAATATCAAAAC<br>CTCCTAAGCAC

Table S6. LncRP11-675F6.3 coding capability analysis by encoding - Coding Potential Calculator (CPC) and coding potential assessment tool (CPAT)

Gene	C/NC	Coding potential Score	
		CPC	CPAT
LncRP11-675F6.3	NC	-1.15822	0.00224

Table S7. RNA pulldown identified proteins using LC-MS/MS

Protein names	Gene names	MW(kDa)
Alpha-actinin-1	ACTN1	102.99263728466
Coatomer subunit beta'	COPB2	102.42248885466
Hexokinase-1	HK1	102.42014361466
Hexokinase-2	HK2	102.31347311466
Staphylococcal nuclease domain-containing protein 1	SND1	101.93351429466
C-1-tetrahydrofolate synthase	MTHFD1	101.49531070466
Stonin-2	STON2	101.10151783466
Methionine--tRNA ligase	MARS	101.05191046466
RNA-binding protein 25	RBM25	100.12441997466
Desmocollin-1	DSC1	99.9237534546602
ATP-dependent RNA helicase DDX54	DDX54	98.5342031146604
Coatomer subunit gamma-1	COPG1	97.6554709546599
Importin subunit beta-1	KPNB1	97.1080192946599
pre-rRNA processing protein FTSJ3	FTSJ3	96.4991989346602
ATP-binding cassette sub-family F member 1	ABCF1	95.8664801946599
Interleukin enhancer-binding factor 3	ILF3	95.2790768446607
Elongation factor 2	EEF2	95.27695382466
Matrin-3	MATR3	94.5648020646601
E3 ubiquitin-protein ligase TRIM71	TRIM71	93.32595295466
Eukaryotic translation initiation factor 3 subunit B	EIF3B	92.4238243846601
Heterogeneous nuclear ribonucleoprotein U	HNRNPU	90.5280091246604

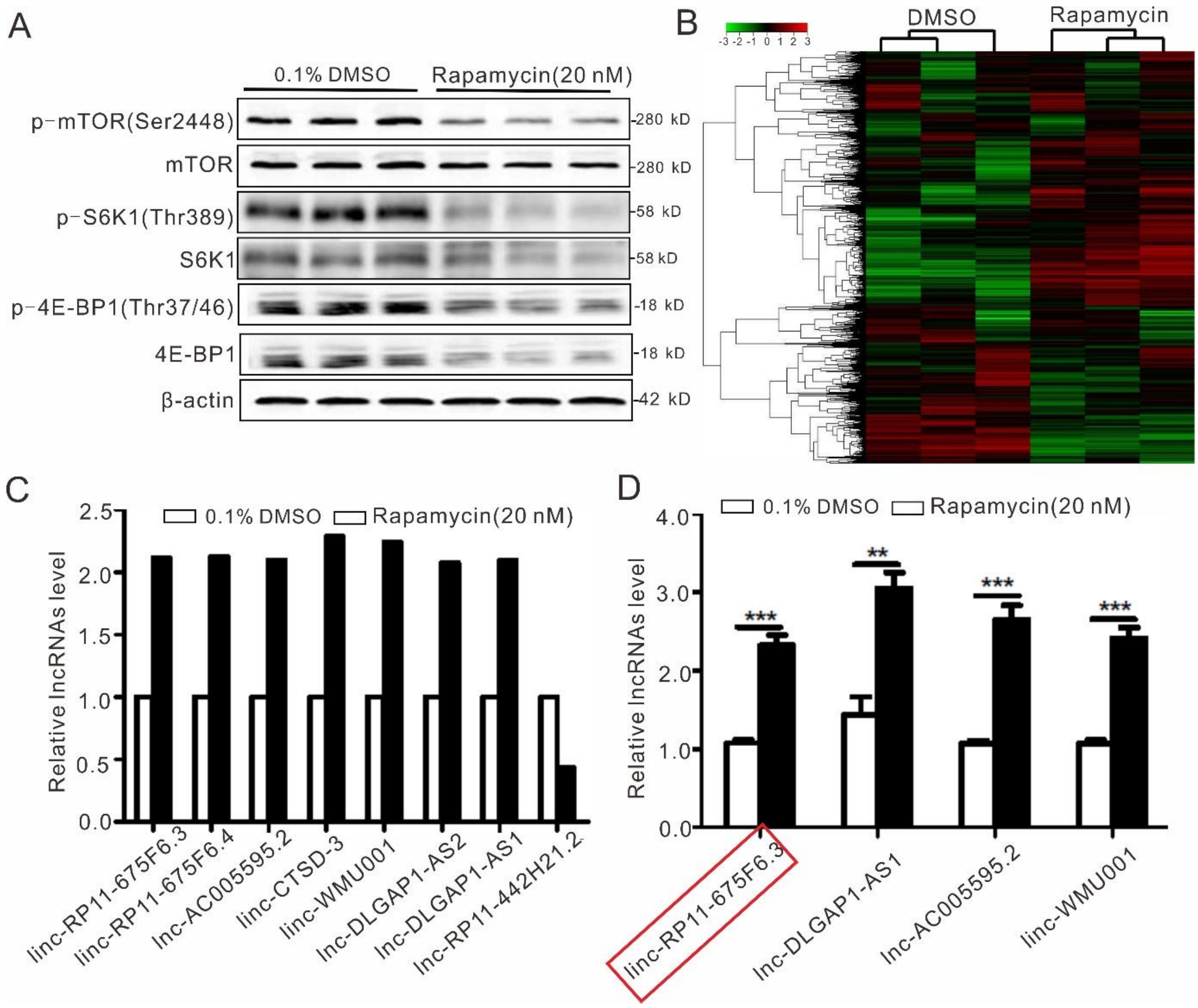

Supplemental Figure S1. Differential expression of LncRNAs in HepG2 cells treated with rapamcyin (A) HepG2 cells were treated with or without rapamycin (20 nM) for 8 h. (B) Two-dimensional hierarchical clustering and scatter plot image of overall lncRNAs differentially regulated in rapamycin treated cells compared with vehicle treated cells. (C) 8 lncRNAs with fold changes >2 in rapamycin treated cells using microarray assay. (D) Validation of differential expression of 4 lncRNAs using RT-qPCR. **$p < 0.01$, ***$p < 0.001$.

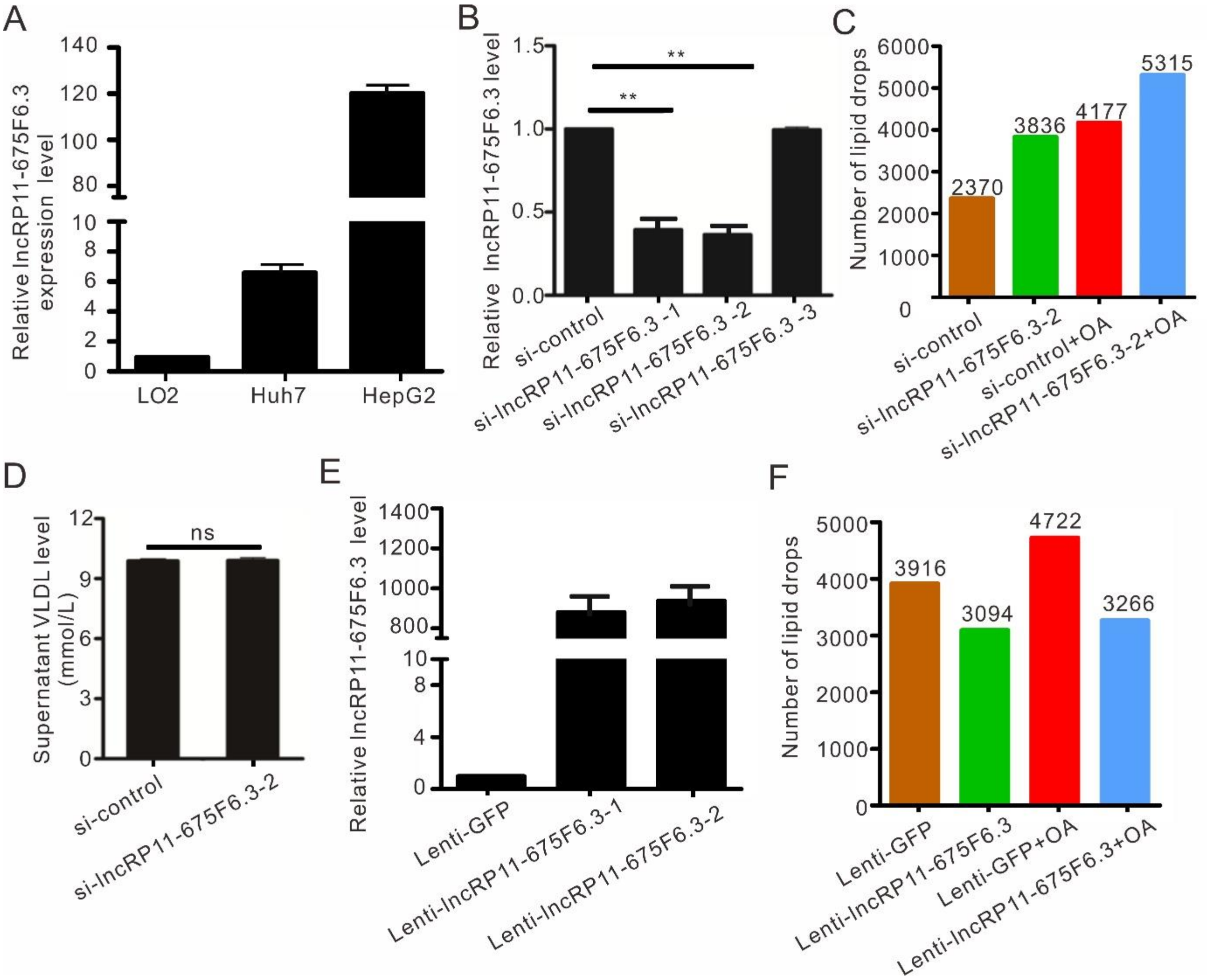

Supplemental Figure S2. (A) Expression of lncRP11-675F6.3 in HL7702 (LO2), Huh7, and HepG2 cells analyzed by RT-qPCR. (B) Relative expression levels of lncRP11-675F6.3 determined by RT–qPCR in HepG2 cells with lncRP11-675F6.3 siRNAs. (C) HepG2 cells treated with si-lncRP11-675F6.3 for 24 h, then added oleic acid (0.4 mmol/L) for 24 h, and Oil Red O staining used to evaluate the number of lipids drops. (D) Knockdown of lncRP11-675F6.3 in HepG2 cells, supernatant VLDL level measured by ELISA. (E) Overexpression of lncRP11-675F6.3 with lentivirus in HepG2 cells confirmed by RT-qPCR, n=3. (F) The number of lipid droplets of Oil Red O staining. Data are expressed as the mean±SEM and analysed by a one-way ANOVA with Tukey's post-hoc test. ns, no significant. ** p<0.01.

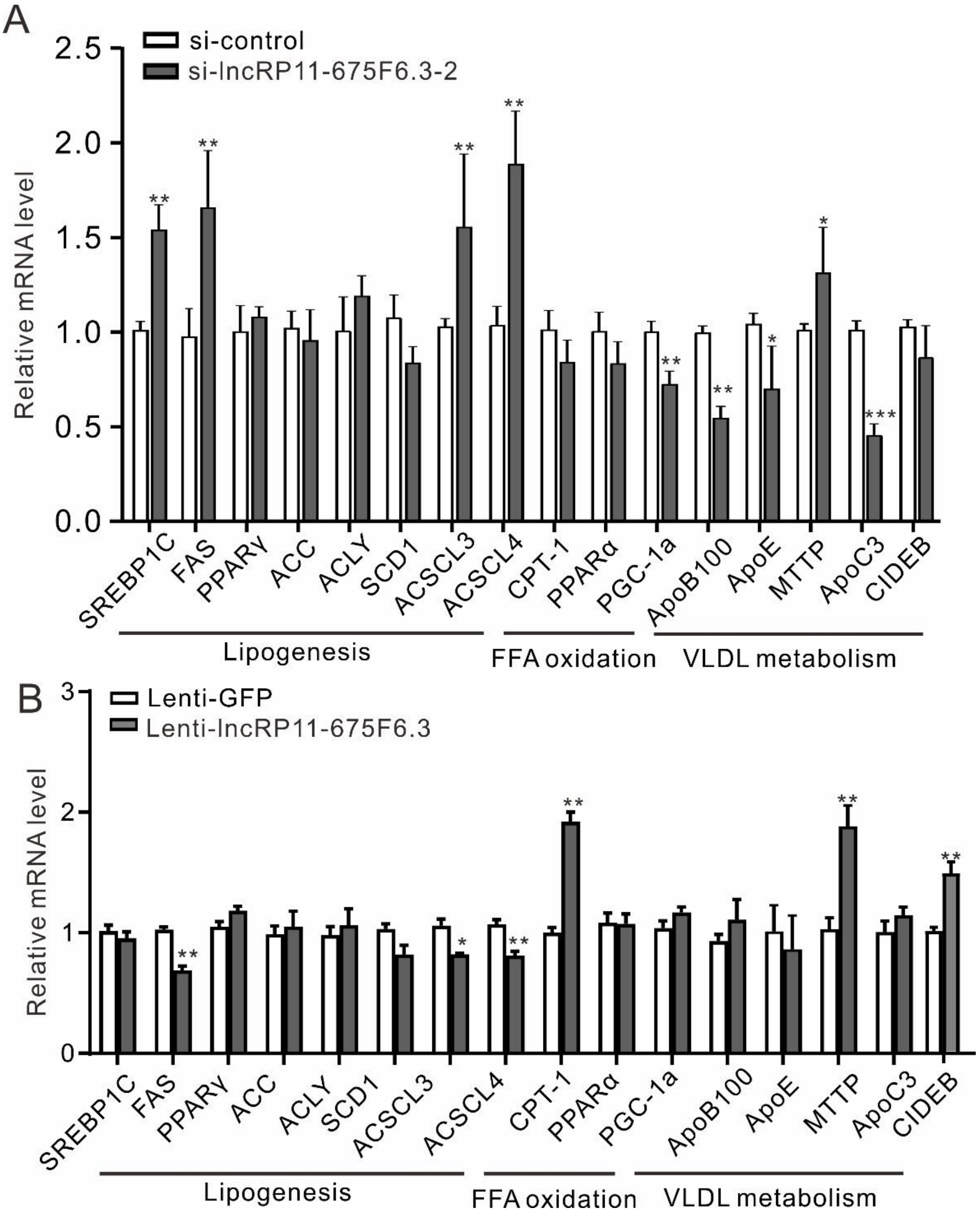

Supplemental Figure S3. LncRP11-675F6.3 affected lipid metabolism related mRNA level. Lipid metabolism relative expression of *SREBP1C, FAS, PPARγ, ACC, ACLY, SCD1, ACSCL3, ACSCL4, CPT-1, PPARα, PGC-1a*, *ApoB100*, *ApoE*, *MTTP*, *ApoC3* and *CIDEB* determined by RT-qPCR in HepG2 cells transfected with lncRP11-675F6.3 siRNAs (A) or overexpression of lncRP11-675F6.3 (B). Data are expressed as the mean±SEM and analyzed by a one-way ANOVA with Tukey's post-hoc test. *$p < 0.05$, ** $p<0.01$.

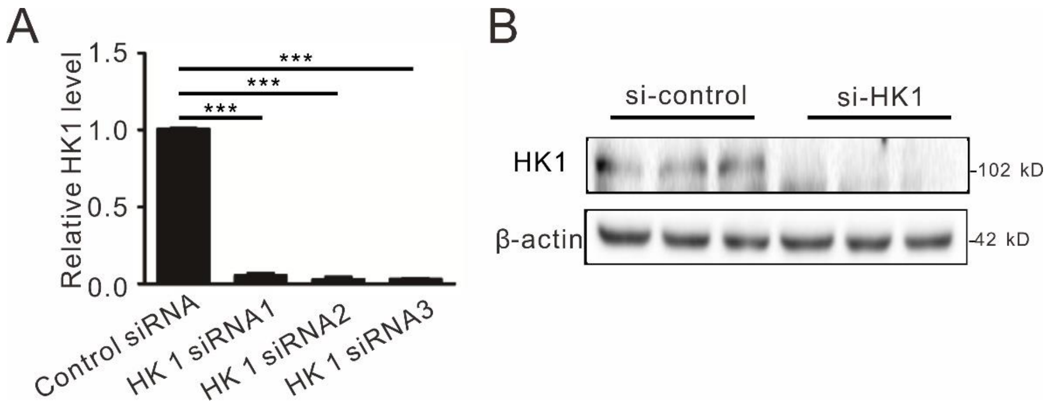

Figure S4. HepG2 cells transfected with HK1 siRNA, then the expression of HK1 measured by RT-qPCR (A) and western blot (B). Data are expressed as the mean±SEM and analyzed by a one-way ANOVA with Tukey's post-hoc test. ***$p < 0.001$.

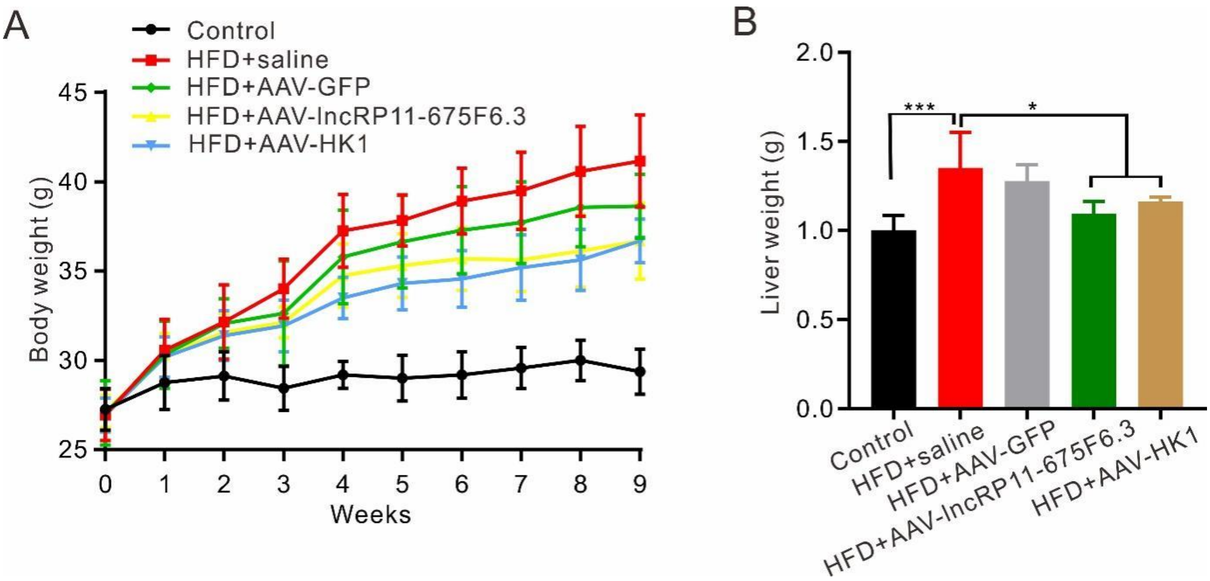

Supplemental Figure S5. LncRP11-675F6.3 and HK1 alleviated lipid accumulation in liver. The mice were feed with high fat died (HFD) for 9 weeks, concurrently mice were injected with adeno-associated virus (AAV) lncRP11-675F6.3 or AAV-HK1 by tail vein once a week (Control group mice were dieted with normal feed). Control group mice were injected AAV-GFP or saline. Every week, the body weight was recorded (A). After 9 weeks, mice was sacrificed and liver weight was detected (B). Data are expressed as the mean±SEM and analyzed by a one-way ANOVA with Tukey's post-hoc test. *$p < 0.05$, ***$p < 0.0001$.
